# Engineering Anti-Tumor Monoclonal Antibodies and Fc Receptors to Enhance ADCC by Human NK Cells

**DOI:** 10.3390/cancers13020312

**Published:** 2021-01-16

**Authors:** Kate J. Dixon, Jianming Wu, Bruce Walcheck

**Affiliations:** Department of Veterinary and Biomedical Sciences, University of Minnesota, Saint Paul, MN 55108, USA; dixon097@umn.edu (K.J.D.); jmwu@umn.edu (J.W.)

**Keywords:** NK cell, ADCC, FcR, CD16, monoclonal antibodies, glycoengineering

## Abstract

**Simple Summary:**

Human natural killer (NK) cells can be targeted to tumor antigens by their IgG Fc receptors that interact with the Fc regions of antibodies that recognize surface proteins on cancer cells. Therapeutic antibodies specific to cancer cell antigens are used to treat various malignancies. NK cells in turn kill antibody-bound tumor cells through a process known as antibody-dependent cell-mediated cytotoxicity (ADCC). The ADCC response of NK cells can be modulated by changes in the antibody or Fc receptor. In this review, we detail the functions of Fc receptors in human NK cells and expand upon current research illustrating how engineering monoclonal antibodies and Fc receptors enhance NK cell-mediated ADCC for the treatment of cancer.

**Abstract:**

Tumor-targeting monoclonal antibodies (mAbs) are the most widely used and characterized immunotherapy for hematologic and solid tumors. The significance of this therapy is their direct and indirect effects on tumor cells, facilitated by the antibody’s antigen-binding fragment (Fab) and fragment crystallizable region (Fc region), respectively. The Fab can modulate the function of cell surface markers on tumor cells in an agonistic or antagonistic manner, whereas the Fc region can be recognized by an Fc receptor (FcR) on leukocytes through which various effector functions, including antibody-dependent cell-mediated cytotoxicity (ADCC), can be elicited. This process is a key cytolytic mechanism of natural killer (NK) cells. These innate lymphocytes in the human body recognize tumor-bound antibodies exclusively by the IgG Fc receptor CD16A (FcγRIIIA). Two allelic versions of CD16A bind IgG with either lower or higher affinity. Cancer patients homozygous for the higher affinity allele of CD16A have been reported to respond significantly better to mAb therapies for various malignancies. These studies revealed that mAb therapy efficacy positively correlates with higher affinity binding to CD16A. Approaches to enhance tumor antigen targeting by NK cells by modifying the Fc portion of antibodies or the FcR on NK cells are the focus of this review.

## 1. Introduction

There are over 100 monoclonal antibodies (mAbs) on the market used as cancer immunotherapeutics that have various mechanisms of action, including checkpoint inhibitors, targeted radiation or toxins, blocking cell growth, and the induction of leukocyte effector functions, among others, that have been described in recent reviews [1,2]. Over 30 Food and Drug Administration-approved mAbs target various tumor antigens. The first mAb therapy utilized for treating cancer, and that continues to be broadly used, is rituximab. This therapeutic mAb recognizes CD20 and has greatly improved the survival rate of patients with B cell malignancies, including non-Hodgkin lymphoma (NHL) and follicular lymphoma [3]. The 4-year overall survival of patients with follicular lymphoma that received the standard chemotherapy of cyclophosphamide, doxorubicin, vincristine, and prednisone (CHOP) was 69%, which increased to 91% for patients that received CHOP with the addition of rituximab (R-CHOP) [3,4]. Antibody-dependent cell-mediated cytotoxicity (ADCC) is an immune process that contributes to the effector mechanisms of rituximab [5]. This cytolytic event is primarily mediated by natural killer (NK) cells, which are lymphocytes of the innate immune system that survey the body for transformed and virally infected cells [6]. ADCC by NK cells is mediated by CD16A, a fragment crystallizable (Fc) receptor (FcR) encoded by *FCRG3A* that binds to IgG [7]. CD16A-expressing NK cells represent the major subset in the peripheral blood (≈90%) and are the focus of efforts to enhance endogenous NK cell-mediated ADCC for clinical applications [8,9]. Multiple studies have been conducted to elucidate the molecular regulation and function of this receptor in NK cells and have led to the initiation of clinical trials centered around exploiting CD16A as a critical NK cell effector molecule [10]. Mechanisms to improve NK cell-mediated ADCC have been shown to enhance the efficacy of cancer therapies that rely on this process for tumor clearance. There have been several approaches aimed at increasing the binding affinity between CD16A and mAbs, including the direct modification of mAbs through amino acid substitutions or glycoengineering [11,12], as well as modifications to the Fc receptor [13,14].

## 2. NK Cells Are Cytotoxic Lymphocytes

**Figure 1:**
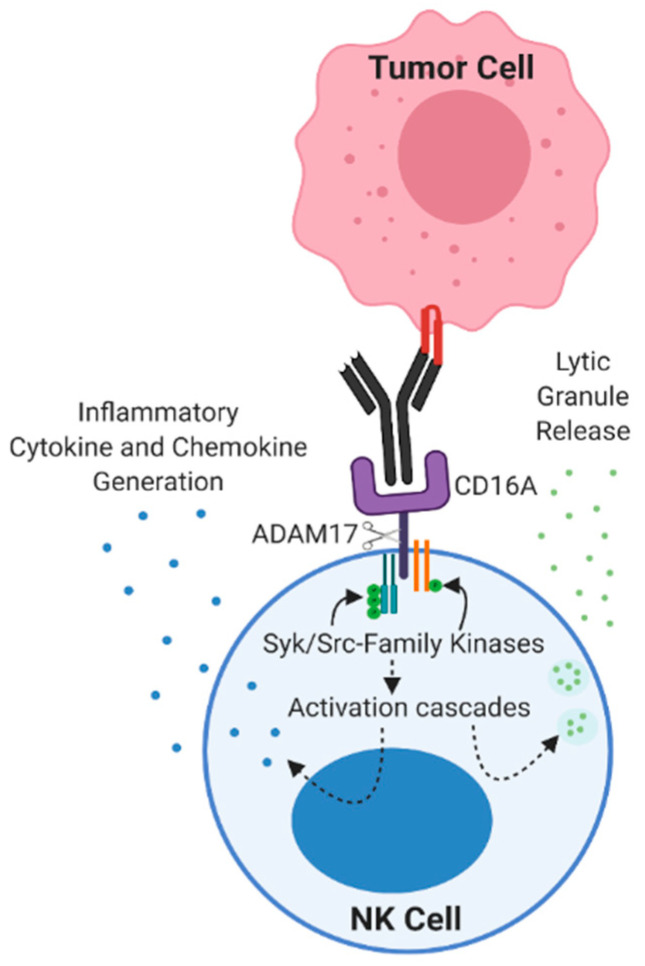
Tumor cell targeting and antibody-dependent cell-mediated cytotoxicity (ADCC) by a natural killer (NK) cell. CD16A binds to tumor-bound IgG resulting in the phosphorylation of CD3ζ (blue) and/or an FcεRIγ chain (orange) by Syk/src family kinases, the induction of various activation cascades, and the release of lytic granules and inflammatory cytokines and chemokines. NK cell activation initiates CD16A cleavage by ADAM17, a regulatory checkpoint that controls CD61A signaling and cell–cell attachment. Figure was created with BioRender.com.

NK cells are the driving immune cell in mediating anti-tumor ADCC during mAb therapy (Figure 1). They comprise 5–20% of circulating lymphocytes in humans and are distinguished by the expression of CD56 and lack of the T cell marker CD3 [6]. NK cells also do not express a T cell receptor and instead interrogate target cells via germline-encoded receptors that detect stress markers (e.g., damage-associated molecular patterns) and the lack of inhibitory markers (e.g., MHC class I proteins). The latter process provides a mechanism of immunosurveillance of tumor cells that have escaped T cell detection [15]. A small subset of circulating NK cells expresses high levels of CD56, referred to as CD56^bright^, and are considered to be immature. These cells primarily release proinflammatory cytokines upon stimulation [6]. Mature NK cells (CD56^dim^) use a variety of activation receptors to directly attach to and kill target cells, a process referred to as natural cytotoxicity. In the presence of cell surface-bound IgG antibodies, NK cells indirectly recognize target cells through an FcR, resulting in ADCC [16]. These cytolytic events are initiated by the formation of a lytic synapse at the interface between an NK cell and the target cell. F-actin in NK cells accumulates at the synapse, lytic granules polarize towards the target cell along a microtubule-organizing center, and this is followed by the exocytosis of their contents, including perforin and granzyme B [17,18,19,20].

## 3. FcγR Expression by Leukocytes

In humans, there are three classes of Fc receptors that bind IgG (FcγRs): FcγRI (CD64), FcγRII (CD32), and FcγRIII (CD16) [21]. FcγR expression by leukocytes induces their activation (CD64, CD32A, CD32C, CD16A, and CD16B) or inhibition (CD32B) to regulate immune responses and signal thresholds [22,23]. Pro-inflammatory and anti-inflammatory cytokines can modulate cell surface levels of the FcγRs [24]. For CD64, the human genome contains three highly homologous *FCGR1* family members (*FCGR1A*, *FCGR1B*, and *FCGR1C*) [25,26]; however, only *FCGR1A* encodes for a functional FcγR, which binds to IgG1 and IgG3 [27,28]. CD64 is expressed constitutively by monocytes, macrophages, and dendritic cells and is upregulated by activated neutrophils. Upon binding immune complexes or antibody-opsonized cells, CD64 induces cell activation by its association with FcεR1γ and is responsible for efficient phagocytosis and antigen presentation [29,30]. As the sole high-affinity FcγR in humans, CD64 binds to monomeric IgG with one to two orders of magnitude higher affinity than the other FcγRs [31,32,33]. The affinity and avidity of CD64 binding to IgG are further increased by inside-out signaling upon leukocyte activation [34,35,36]. For instance, during the cytokine stimulation of neutrophils or monocytes, CD64 formed increased clusters in the cell membrane by a process that involved cytoskeletal rearrangement and protein phosphatase 1 (PP1) [36].

*FCGR2A*, *FCGR2B*, *FCGR2C, FCGR3A*, and *FCGR3B* encode for low- to medium-affinity FcγRs that bind to IgG1–4 [37]. *FCGR2A, FCGR2B,* and *FCGR2C* encode for CD32A, CD32B, and CD32C, which are broadly expressed by most human immune cells and platelets. All three of the CD32 proteins are integral membrane glycoproteins and share 85% amino acid homology [38]. While CD32B possesses inhibitory functions, CD32A and CD32C are well-characterized activating receptors [39]. CD32A and CD32C activation signaling is mediated by an immunoreceptor tyrosine-based activation motif (ITAM) sequence in their cytoplasmic domain [39]. Interestingly, the CD32A ITAM is non-canonical and contains an additional three aspartic acid residues [40]. CD32A activation mediates phagocytosis by neutrophils, monocytes, and macrophages [39,41]. In contrast, CD32B contains an immunoreceptor tyrosine-based inhibition motif (ITIM) that acts to counter ITAM-mediated activation signaling [39]. The activation of CD32B in response to the engagement of IgG recruits the SH2 domain-containing inositol 5-phosphatase (SHIP), resulting in the dephosphorylation of numerous activation substrates [42]. CD32B associates with the B cell receptor on activated B cells and downregulates IgG production. This is an important feedback loop for sensing soluble IgG and shutting down IgG secretion while soluble IgG levels are high [42]. Due to the high sequence homology between CD32A and CD32C, the latter has been difficult to study [37]. Further, the *FCGR2C* gene is subject to a stop polymorphism and only 7–15% of the population expresses a functional CD32C protein [43]. The expression of CD32C has been reported in NK cells and it may enhance CD16A signaling and the induction of ADCC [44].

*FCGR3A* and *FCGR3B* encode for CD16A and CD16B [37,45]. In addition to its expression by human NK cells, CD16A is also expressed on γδ T cells and subsets of monocytes/macrophages [22,46], whereas CD16B is primarily expressed by neutrophils [47]. CD16A and CD16B share 97% amino acid homology in their extracellular region but differ considerably in their means of membrane attachment. CD16A is a type I transmembrane protein, while CD16B is attached by glycosylphosphatidylinositol (GPI) linkage. The IgG-binding affinity by CD16A is marginally higher than CD16B and is modulated by one residue, Gly129 [48]. Mutation of this residue in CD16A reduced its IgG-binding affinity and the conversion of Asp129 in CD16B to a Gly increased its binding affinity [48].

### 3.1. CD16 Signaling

CD16A and CD16B are potent activating receptors, yet they vary markedly in their means of cell signaling. CD16B associates with complement receptor 3 (CR3, α_M_β_2_, CD11b/CD18, and Mac-1) or complement receptor 4 (CR4, α_X_β_2_, and CD11c/CD18) for signal transduction, inducing phagocytosis and the consequent respiratory burst [49,50,51,52]. CD16A, on the other hand, associates in a noncovalent manner with homo- or heterodimers of FcεR1γ and/or CD3ζ [53], which contain one or three ITAMs, respectively [21]. Studies indicate that there are functional differences in human NK cell subsets based on CD16A association with FcεR1γ or CD3ζ. For instance, adaptive NK cells occurring in individuals previously infected by human cytomegalovirus (CMV) mediate CD16A signaling primarily through CD3ζ and demonstrate increased cytokine production and ADCC [54,55]. CD16A engagement of FcεR1γ and CD3ζ leads to the activation of kinases Syk, zeta chain-associated protein kinase-70 (ZAP-70), and lymphocyte-specific protein tyrosine kinase (Lck), and the induction of the phosphoinositide 3-kinase (PI3K), Vav1, and phospholipase C gamma (PLCγ) signaling cascades [56,57,58,59]. PLCγ hydrolyzes PIP2 into IP3 and diacylglycerol (DAG). IP3 induces a calcium flux and subsequently activates the phosphatase calcinerin, which dephosphorylates nuclear factor of activated T cells (NFAT) and allows for NFAT nuclear translocation, while DAG activates protein kinase C (PKC) [60]. Collectively, NFAT and PKC induction leads to several downstream activation effects, resulting in the degranulation and the generation of proinflammatory cytokines [61]. The PI3K pathway activates the MAP kinase cascade of Rac1→MEK→ERK, which polarizes actin and microtubule organization, allowing for effective granzyme B and perforin release [62]. While the membrane-proximal signaling molecules associated with the induction of ADCC via CD16A have been established, the downstream signaling factors involved remain an active area of research. It has been shown that when NK cells are stimulated through CD16A, 21 different kinases are phosphorylated, and this list includes known proteins downstream of CD16A, such as Lck, as well as kinases not previously associated with CD16A activation, such as FAK2 and Fyn [63]. However, there are redundancies in NK cell activation signaling during ADCC. For instance, ZAP-70 phosphorylation has been well studied downstream of CD16A [64,65], yet ZAP-70-deficient NK cells can still mediate ADCC [66]. Taken together, CD16A upon the engagement of cell-bound antibodies induces robust NK cell activation that is sufficient for cytotoxicity and cytokine generation, whereas other activation receptors in these cells, some of which also couple with ITAM adaptors, require co-stimulation [67].

### 3.2. Natural Polymorphisms in CD16A Increase IgG Binding Affinity

Genetic polymorphisms affect the affinity by which CD16A binds to IgG [68,69,70]. A polymorphism at amino acid position 158 is a result of a G to T point mutation at nucleotide 559 that results in a valine (Val or V) to phenylalanine (Phe or F) substitution [69,71]. Polymorphisms have also been reported at amino acid position 48 that result in leucine (Leu), arginine (Arg), or histidine (His) expression [69,70]. Polymorphisms at position 48 are linked to polymorphisms at position 158, with 48Leu correlating with 158Phe (158F), and 48Arg and 48His to 158Val (158V) [69]. However, the increased affinity for IgG is independent of residue 48 polymorphisms alone [72]. Polymorphisms at amino acid 158 change affinity for IgG1, with CD16A 158V binding IgG at a ~2 fold higher affinity than CD16A 158F [32], yet the latter is the dominant allele in humans [73]. Studies have shown that patients with non-Hodgkin lymphoma who are treated with the anti-CD20 mAb rituximab show improved clinical responses when homozygous for a valine substitution at position 158 [74]. In addition, the treatment of cancers with cetuximab (anti-EGFR) or trastuzumab (anti-HER2) results in higher response rates among patients with the CD16A 158V polymorphism [9,75].

## 4. Therapeutic mAb Engineering

Given that increased IgG binding affinity due to CD16A polymorphisms can enhance ADCC and improve clinical outcomes, a strategy has emerged to improve therapeutic mAb function by modifying the Fc portion of tumor-targeting antibodies. This has been achieved through different approaches, such as glycosylation and amino acid substitutions. Shields et al. performed alanine screening on the Fc region of IgG1 and found that the combined substitution of Ser298, Glu333, and Lys334 to alanine increased binding affinity to CD16A and resulted in augmented ADCC [11]. Other point mutations have also been shown to be effective at increasing IgG binding affinity to CD16A, including Ser239Asp and Ile332Glu, as well as Gly236Ala, Ser239Asp, Ala330Leu, and Ile332Glu (referred to as GASDALIE) [76,77]. The GASDALIE mutations increased the affinity of IgG binding to CD16A by 20-fold, while only slightly increasing binding affinity to the inhibitory FcγR CD32B [76]. Increasing affinity for CD16A while not increasing the affinity for CD32B, which could dampen activation signaling, is expected to increase leukocyte effector functions. The increase in CD16A affinity was also observed with a similar series of mutations: Ser239Asp, Ala330Leu, and Ile332Glu (SDALIE) [76,78,79]. An anti-FMS-related tyrosine kinase 3 (FLT-3) mAb with Ser239Asp and Ile332Glu mutations is part of a clinical phase I/II trial to treat patients with acute myeloid leukemia (AML) (ClinicalTrials.gov identifier: NCT02789254). Recent in vitro studies using this FLT-3 mAb suggested that it could also be suitable for the treatment of B cell acute lymphoblastic leukemia (B-ALL) [80]. A screen that aimed to elucidate increased CD16A binding/decreased off rate and also prevent increased affinity for inhibitory CD32B found that the combination of Phe243Leu, Arg292Pro, Tyr300Leu, Val305Ile, and Pro396Leu (referred to as Variant 18) achieved this [81]. The anti-HER2 mAb margetuximab contains the Variant 18 mutations and has shown promise in clinical trials, with 78% of patients evaluated showing reduced tumor size [82]. Margetuximab showed greater progression-free survival (PFS) than the non-modified therapeutic mAb trastuzumab in a phase III clinical trial for treating HER2-positive breast cancer patients (NCT02492711) [83]. These clinical outcomes demonstrate a functional benefit from increasing the affinity between mAbs and CD16A.

Glycosylation is a post-translational protein modification occurring in the endoplasmic reticulum and Golgi apparatus that regulates the structure and function of many proteins [84]. There are two primary types of linkages for oligosaccharides, N-linked and O-linked. N-linked glycans tend to be large and branched and are initiated by an N-acetylglucosamine (GlcNAc) that is attached to the nitrogen of an asparagine. N-linked glycans are added to a conserved Asn297 in the Fc region of human IgG. Structural NMR studies have shown that glycosylation alters the C’E loop which is located in the Cγ2 domain of the Fc region of IgG and this affects its binding affinity to CD16A [12]. Different types of N-glycan modifications occur, though all share a common backbone with two N-acetylglucosamine units followed by three mannose moieties [85]. An important N-glycan decoration is a core fucosylation (α1,6) at the first GlcNAc [86]. Removal of this fucose increases IgG binding affinity to FcγRs and ADCC by NK cells [87,88,89]. These studies reveal that the glycosylation of IgG1 has an allosteric impact on CD16A ligation but is not directly involved in CD16A binding [12].

### Glycoengineered Anti-CD20 mAb Enhances NK Cell-Mediated ADCC

Anti-CD20 mAbs are classified as type I or type II based on glycosylation, function, and their direct biological effects. Type I mAbs, such as rituximab, induce lipid raft formation upon binding to CD20, which triggers calcium flux and caspase-mediated apoptotic signaling [90,91]. While type II mAbs also induce apoptosis, they do so in a lysosome-mediated, caspase-independent manner [92]. Obinutuzumab is a type II glycoengineered mAb that lacks the core fucose moiety on the first N-acetylglucosamine and was developed specifically to address issues of rituximab resistance in cancer patients [93,94,95]. Interestingly, a non-glycoengineered version of obinutuzumab and F(ab’)_2_ fragments of this mAb induced the same level of apoptosis when added to Raji cells (a B cell lymphoma cell line) as the glycoengineered antibody, suggesting that this specific function is Fc independent [92]. Type I mAbs demonstrate higher binding levels at saturating conditions compared to type II antibodies. Stoichiometry studies have shown that type I antibodies bind CD20 in a 1:1 or 2:1 (IgG:CD20) ratio, forming “seeding complexes” that enable further binding of IgG to CD20 molecules, whereas type II mAbs bind to CD20 in a 1:2 ratio, forming “terminal complexes” that do not allow the binding of additional IgG molecules [96]. This difference in numerical relationship between antibody and antigen appears to account for increased ADCC induction by type II anti-CD20 mAbs, especially in patients expressing the lower affinity CD16A (158F) variant [95]. Indeed, while obinutuzumab and rituximab both bind the same epitope on CD20, obinutuzumab demonstrated increased NK cell-dependent ADCC and IFNγ generation [97,98]. Moreover, obinutuzumab showed prolonged PFS and higher minimum residual disease (MRD) negative outcomes compared to rituximab in chronic lymphocytic leukemia (CLL) patients [99].

## 5. FcR Modifications Through NK Cell Engineering

A downside to Fc engineering of anti-tumor therapeutic mAbs is that the modification of individual antibodies to enhance lymphocyte effector functions involves considerable work and high production costs, especially given the time and resources required to test the safety and efficacy of every mAb that is modified [100]. An alternative approach is modifying CD16A in human NK cells. There are several ways this is being investigated. For instance, CD16A is cleaved through a process referred to as ectodomain shedding by a disintegrin and metalloproteinase-17 (ADAM17) (Figure 1) [13,101,102,103]. ADAM17 is a transmembrane metalloprotease that is expressed on the surface of NK cells and cleaves CD16A in a *cis* manner upon cell activation by multiple stimuli [104]. Targeting ADAM17 activity with either a small molecule inhibitor or blocking mAb resulted in increased IFN-γ generation during ADCC [102,105]. Additional means of targeting ADAM17 activity have used CRISPR/Cas9 to knock out this sheddase in peripheral blood NK cells, which resulted in increased IFN-γ generation and ADCC by these cells when combined with rituximab-opsonized Raji target cells [106].

Blocking CD16A shedding can also be achieved by modification of the ADAM17 cleavage site in CD16A. ADAM17 cleaves CD16A at an extracellular location proximal to the cell membrane between residues Ala195 and Val196 [13,103]. Substituting an adjacent serine at position 197 for a proline renders CD16A resistant to ADAM17 cleavage, referred to as non-cleavable CD16A (ncCD16A) [13]. This mutation has been engineered into the higher affinity (158V) variant of CD16A to increase the affinity and avidity at which the FcγR binds to antibody-opsonized tumor cells. Non-cleavable CD16A expressed in NK92 cells (a human NK cell line) showed enhanced IFN-γ generation during the ADCC of trastuzumab-opsonized SKOV3 cells (ovarian cancer cell line) [105]. When ncCD16A was expressed in primary human NK cells derived from induced pluripotent stem cells (iPSCs), these cells mediated higher levels of ADCC and cytokine production than peripheral blood NK cells in in vitro assays when co-cultured with various therapeutic mAbs and tumor types, including Burkitt’s lymphoma, lung adenocarcinoma, ovarian adenocarcinoma, and squamous cell carcinoma cells [107]. In addition, iPSC-derived NK cells expressing ncCD16A administered to mice in human lymphoma or ovarian cancer xenograft models showed increased tumor regression and enhanced survival [107]. The utilization of the ncCD16A receptor by Fate Therapeutics (referred to as FT516) is being employed in the treatment of acute myeloid leukemia in a phase I clinical trial (NCT04023071), as well as in a phase I trial as a therapeutic for patients hospitalized with hypoxia from COVID-19 (NCT04363346). Clinical trials are also ongoing with iPSC-derived NK cells expressing ncCD16A and an anti-CD19 chimeric antigen receptor (CAR), as a combination targeting approach for the treatment of B-cell lymphoma (BCL) and CLL (NCT04245722).

Other approaches utilizing the higher affinity CD16A variant evaluate its function in human NK cell lines. NK92 cells that have been modified to express CD16A 158V and the self-production of IL-2 (referred to as high-affinity NK cells or haNK cells) showed potent ADCC against target cells with several mAb treatments in vitro [108]. Jochems et al. showed that irradiated haNK cells were able to successfully induce ADCC in the presence of the checkpoint inhibitor anti-PD-L1 antibody avelumab in a phase II clinical trial to treat Merkel cell carcinoma (NCT03853317) [14].

While NK cell products expressing CD16A 158V have been referred to as expressing a “high-affinity” FcγR, this CD16A variant binds IgG with moderate affinity relative to CD64, the only high-affinity FcγR [21,32]. CD64 binds to the same IgG isotypes as CD16A (IgG1 and IgG3) but with >30-fold higher affinity [32,33]. Unlike CD16A, CD64 is not downregulated on the surface of leukocytes by ADAM17 upon their activation [109]. Work in our lab has focused on the use of recombinant versions of CD64 expressed in engineered NK cells to increase their ADCC potency [110]. NK92 cells and iPSC-derived NK cells expressing a recombinant receptor containing the extracellular portion of CD64 with the transmembrane and intracellular region of CD16A (referred to as CD64/16A) can kill mAb-opsonized tumor cells in vitro via ADCC better than NK cells expressing the higher affinity CD16A variant [109]. Knowledge obtained from current studies that are optimizing NK cell signaling domains in CAR constructs could be applied to enhance the function of recombinant CD64 [111]. For instance, Li et al. found that when comparing different transmembrane and intracellular signaling domains in iPSC-derived NK cells, an anti-mesothelin CAR with an NKG2D transmembrane region plus 2B4 and CD3ζ signaling domains increased their cytotoxicity and persistence in vivo [111]. CD16A or CD64 with exogenous co-stimulation domains would signal differently from the native receptors in terms of pathways and kinetics. CD16A signals by association with the signaling adapters CD3ζ and FcεR1γ, as described above, whereas the exogenous signaling domains would bypass the involved stoichiometry of these intermolecular interactions. This may accelerate the induction of NK cell activation and formation of the lytic synapse, as well as alter downstream signaling effectors that modulate cell proliferation and cytokine production.

## 6. Conclusions

**Figure 2:**
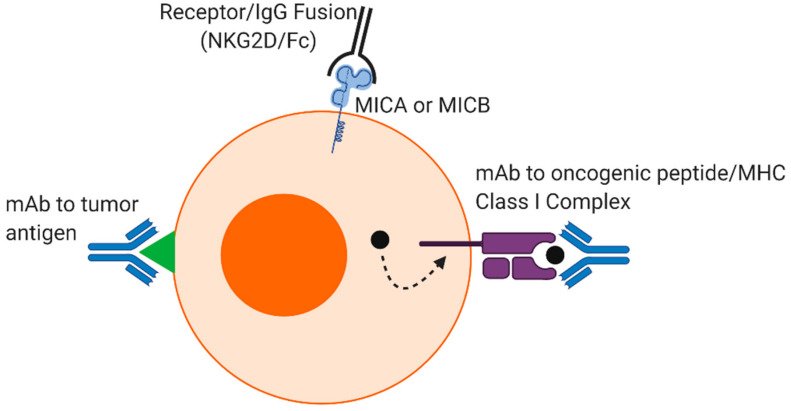
Recognition of tumor cells by monoclonal antibodies (mAbs). Therapeutic mAbs can recognize tumor antigens and oncogenic peptide/MHC class I complexes on malignant cells. Receptor/IgG fusion proteins, such as NKG2D/Fc, target tumor-associated ligands MICA or MICB.

In summary, therapeutic mAbs are one of the largest classes of modern biopharmaceuticals and continue to grow each year [112,113,114]. In addition to recognizing cell surface tumor antigens, mAbs can be used to recognize intracellular tumor antigens such as oncogenic peptide/MHC class I complexes [115]. Tumor-associated markers can also be detected by receptor–IgG chimeric proteins, such as NKG2D-IgG that binds MICA and MICB, ligands that are upregulated in various malignancies [116]. These products provide an ever-increasing repertoire of tumor-associated and tumor-specific targeting elements (Figure 2). Recent approaches have attempted to address mechanisms of resistance to mAb therapy by modification of the Fab and Fc regions. For the latter, this has involved various types of Fc engineering to change glycosylation and amino acid residues to enhance ADCC. An alternative approach to the optimization of individual mAbs is the engineering of NK cells with enhanced FcγRs.

Preclinical and clinical studies have shown that adoptive autologous and allogeneic NK cell therapies are safe and that the latter are less likely to evoke GVHD (graft versus host disease) and other adverse events compared to T cells [117,118,119]. NK cells expressing transgenic receptors can also recognize tumor ligands through native germline receptors, reducing tumor cell escape. A limitation of NK cell therapy is that they are very heterogenous and this creates challenges for standardization and multiple-dosing strategies [120,121,122,123,124]. NK cell lines address these issues and are being used in clinical trials, but they are transformed cells and must be mitotically inactivated before infusion into patients, and thus these treatments require multiple administrations. Our approach has focused on the use of iPSCs for NK cell generation. iPSCs are amenable to genetic modification and the derived NK cells can be produced in a clonal and clinically scalable manner [125,126]. Moreover, these cells demonstrate similar phenotypic markers and anti-tumor effector functions as peripheral blood NK cells [127]. High-affinity FcγR-modified iPSC-derived NK cells have the potential to be a promising cancer immunotherapy by allowing for universal tumor antigen targeting by mAb therapies. As the mutation-driven resistance of tumors is further studied, immunotherapies and targeted drugs will offer diverse combinatorial options to prevent tumor escape variants and achieve a durable cure [128,129].
